# Supplementary material for: Critical scaling of whole-brain resting-state dynamics
Source: Commun Biol. 2023 Jun 10;6:627. doi: 10.1038/s42003-023-05001-y (PMC10257708; doi:10.1038/s42003-023-05001-y)
Supplement: Supplementary file 2 — Supplementary Information [file 42003_2023_5001_MOESM2_ESM.pdf]

Supplementary information

Critical scaling of whole-brain resting-state dynamics

Adrián Ponce-Alvarez, Morten L. Kringelbach, Gustavo Deco.

Supplementary figures

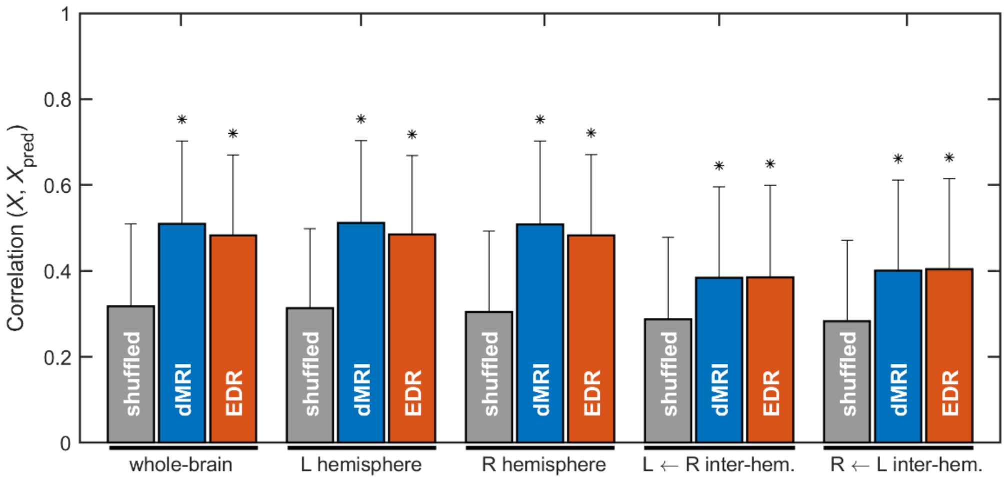

Supplementary figure S1. In addition to calculating the linear prediction of fMRI signals for the whole brain (see Figure 1d), we also computed it separately for each brain hemisphere, and for one hemisphere given the activity of the other hemisphere and the inter-hemispheric connections. Specifically, we computed the linear prediction of the left hemisphere's activity $\boldsymbol{X}^{(L)}$ given the corresponding intra-hemispheric connectivity $\boldsymbol{C}^{(L,L)}$, i.e., $\boldsymbol{X}_{\text{pred}}^{(L)} = \boldsymbol{C}^{(L,L)}\boldsymbol{X}^{(L)}$, and analogously for the right hemisphere, i.e., $\boldsymbol{X}_{\text{pred}}^{(R)} = \boldsymbol{C}^{(R,R)}\boldsymbol{X}^{(R)}$. We also computed the linear prediction of the left hemisphere's activity $\boldsymbol{X}^{(L)}$ given the inter-hemispheric connectivity $\boldsymbol{C}^{(L,R)}$ and the activity of the right hemisphere $\boldsymbol{X}^{(R)}$, i.e., $\boldsymbol{X}_{\text{pred}}^{(L)} = \boldsymbol{C}^{(L,R)}\boldsymbol{X}^{(R)}$, and analogously for the right hemisphere, i.e., $\boldsymbol{X}_{\text{pred}}^{(R)} = \boldsymbol{C}^{(R,L)}\boldsymbol{X}^{(L)}$. The connectivity matrix $\boldsymbol{C}$ was given by the dMRI, the EDR, or a shuffled connectivity that preserves the distribution of dMRI weights but destroys their spatial organization. The goodness of the linear prediction was given by the Pearson correlation between $X_{\text{pred}}^{(j)}(t)$ and $X^{(j)}(t)$, where $j = L$ or R, for all nodes and all subjects. Bars indicate the average goodness of the linear predictions and error bars represent the SD. Asterisks indicate predictions that were significantly better ($p < 0.001$, Welch's *t*-test after Fisher z-transformation) than the one obtained using the shuffled connectivity. We found that, although inter-hemispheric predictions were reduced with respect to intra-hemispheric ones (dMRI: 0.51 vs. 0.39; EDR: 0.48 vs. 0.39), they remained significant ($p < 0.001$) and were practically indistinguishable using the dMRI and the EDR connectivity matrices (0.39).

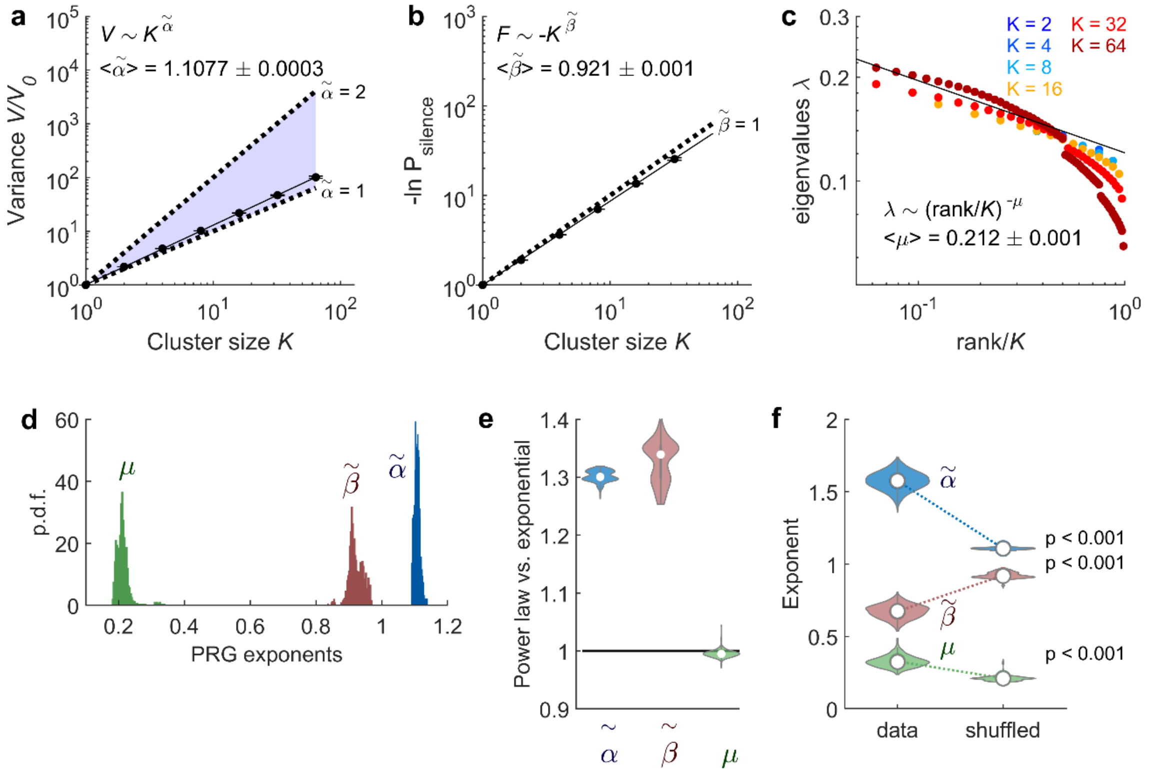

Supplementary figure S2. For each subject, shuffled data were built by randomly permuting the time frames of binarized fMRI signals for each ROI separately. Next, the PRG method was applied to the resulting shuffled data. **a)** Variance V of coarse-grained variables as a function of cluster size K, average over subjects (black points; error bars indicate SD over subjects). The solid black line indicates least squares power law fit, i.e. $V = K^{\tilde{\alpha}}$. Dashed lines indicate linear ($\tilde{\alpha} = 1$) and quadratic ($\tilde{\alpha} = 2$) growths, corresponding to uncorrelated and fully correlated systems, respectively. $\langle\tilde{\alpha}\rangle$ indicates the average exponent across subjects. **b)** Silence log-probability, $F = \ln P_{\text{silence}}$, of coarse-grained variables as a function of cluster size, average over subjects (black points; error bars indicate SD over subjects). The solid black line indicates least squares power law fit, i.e. $F = -K^{\tilde{\beta}}$. The dashed line indicates the prediction for uncorrelated variables ($\tilde{\beta} = 1$). $\langle\tilde{\beta}\rangle$ indicates the average exponent across subjects. In (a) and (b), the variance and the silence log-probability were normalized by their corresponding values at coarse-graining step $k = 0$ (original system). **c)** Eigenvalues λ of the covariance matrix as a function of their relative rank, for clusters of different sizes, for one example subject. The solid black line indicates least squares power law fit, i.e. $\lambda = (\text{rank}/K)^{-\mu}$. $\langle\mu\rangle$ indicates the average exponent across subjects. **d)** Distribution of exponents $\tilde{\alpha}$, $\tilde{\beta}$, μ for single-subject scans. **e)** Distribution of ratios between the explained variances of power-law and exponential fits (R_{EV}). Note that for most of the individual shuffled scans the eigen-spectrum showed no evidence of power-law scaling. **f)** Comparison between exponents obtained using the original data and the shuffled data. The p-value of Wilcoxon tests comparing exponents is also indicated.

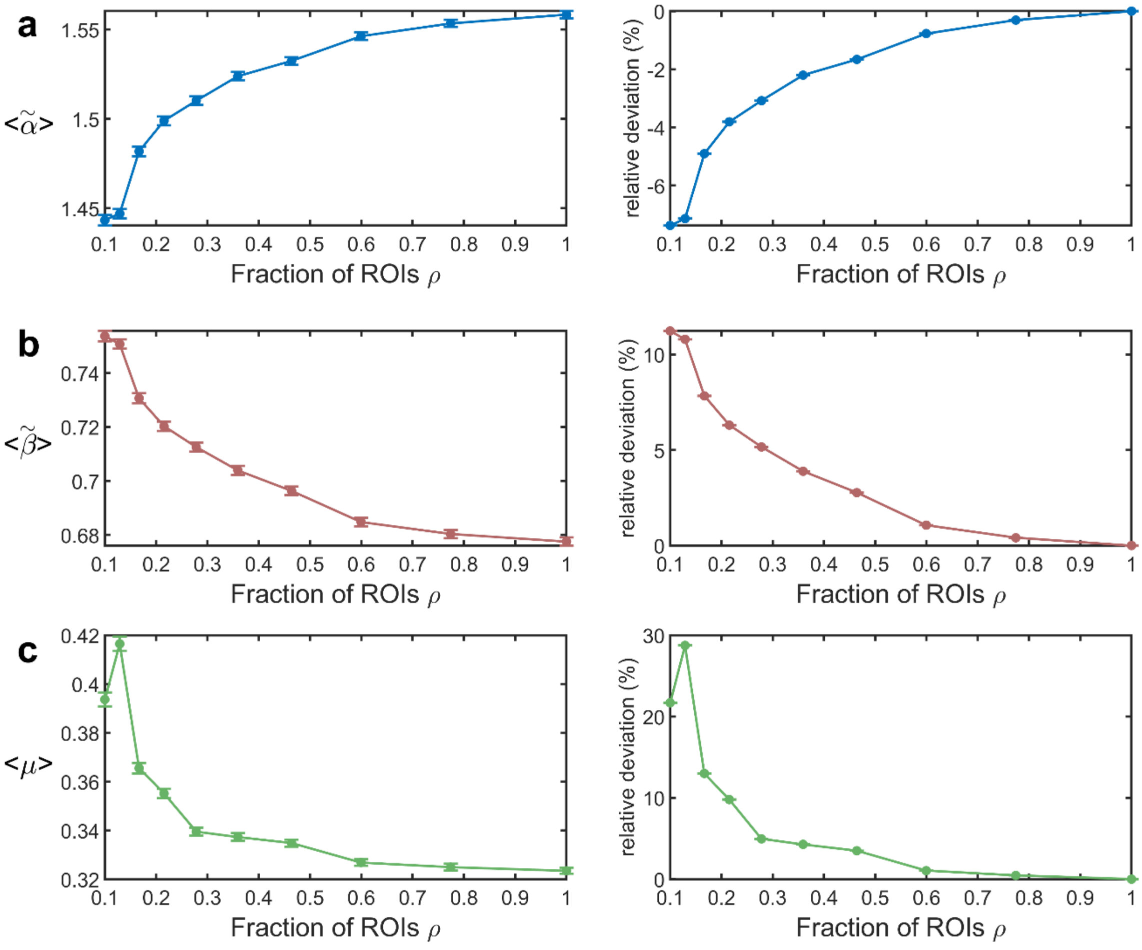

Supplementary figure S3. For each subject, subsampled systems were built by randomly selecting a fraction ρ of the N ROIs. Next, the PRG method was applied to the subsampled data. **a-c)** *Left:* PRG exponents, average across subjects, as a function of the fraction of ROIs. Error bars indicate SEM. *Right:* relative deviation from the exponent value obtained from the full-size system, i.e., $100\times\left(x(\rho)-x(\rho=1)\right)/x(\rho=1)$, where x denotes the exponent. Note that PRG exponents from subsampled data converged (absolute relative deviation < 1%) to those obtained using the full-size original data (i.e., $\rho=1$) when the fraction of selected ROIs was larger than $\rho{\sim}0.7N$.

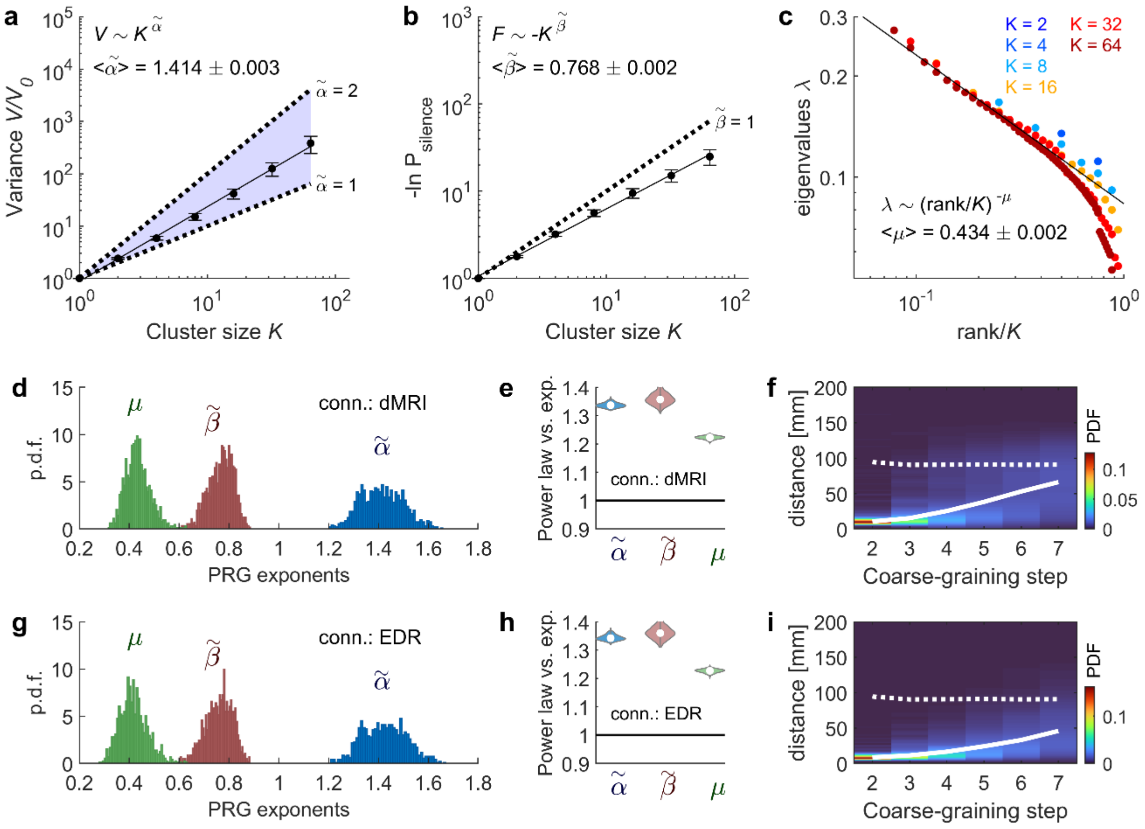

Supplementary figure S4. Connectivity-based PRG. The fMRI activity was coarse-grained based of the structural connectivity, instead of the functional correlations (see Methods). **a)** Variance V of coarse-grained variables as a function of cluster size K, average over subjects (black points; error bars indicate SD over subjects). The solid black line indicates least squares power law fit, i.e. $V = K^{\tilde{\alpha}}$. $\langle\tilde{\alpha}\rangle$ indicates the average exponent across subjects. **b)** Silence log-probability, $F = \ln P_{\text{silence}}$, of coarse-grained variables as a function of cluster size, average over subjects (black points; error bars indicate SD over subjects). The solid black line indicates least squares power law fit, i.e. $F = -K^{\tilde{\beta}}$. $\langle\tilde{\beta}\rangle$ indicates the average exponent across subjects. **c)** Eigenvalues λ of the covariance matrix as a function of their relative rank, for clusters of different sizes, for one example subject. The solid black line indicates least squares power law fit, i.e. $\lambda = (\text{rank}/K)^{-\mu}$. $\langle\mu\rangle$ indicates the average exponent across subjects. **d)** Distribution of exponents $\tilde{\alpha}$, $\tilde{\beta}$, μ for single-subject scans. **e)** Distribution of ratios between the explained variances of power-law and exponential fits (R_{EV}). **f)** Colormap: distribution of distances between grouped nodes as a function of coarse-graining. The solid white trace indicates the median distance. The dashed white line indicates the median distance in the case of shuffled connectivity. In panels (a)-(f) the coarse-graining was based on the dMRI connectivity. **g-i)** Same as (d), (e), and (f) but for the EDR-based PRG.

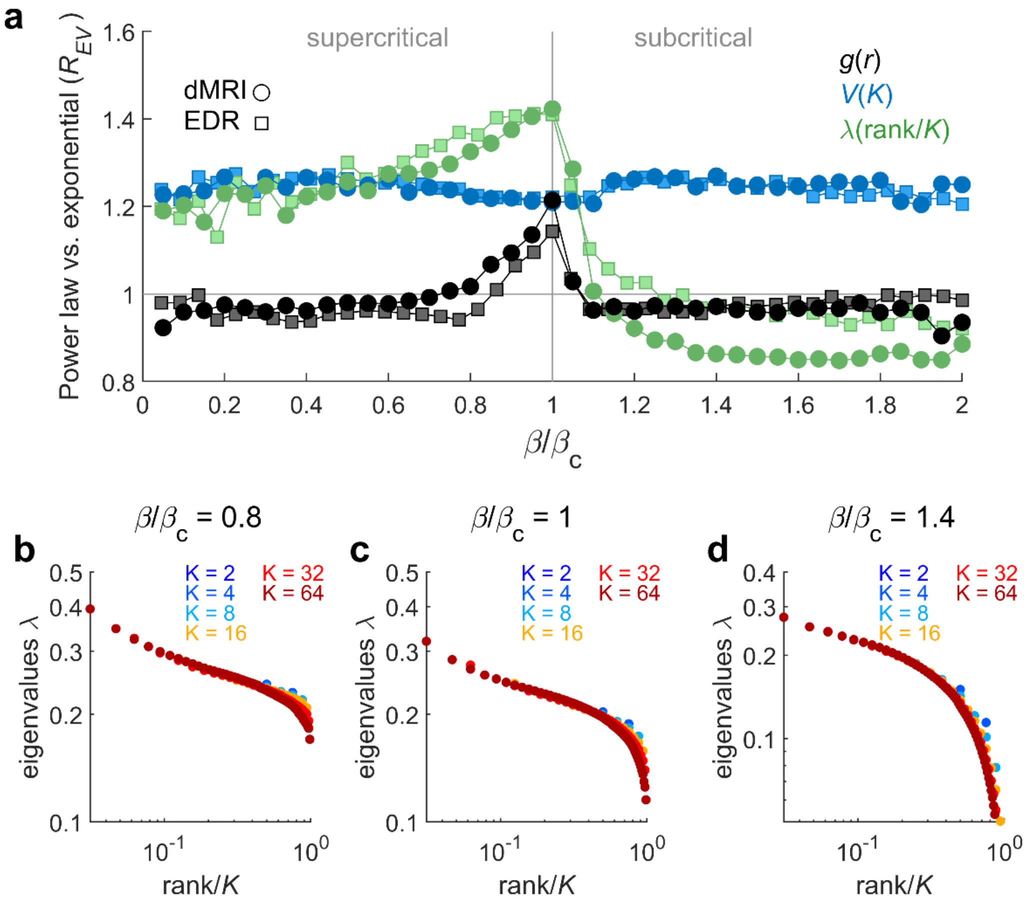

Supplementary figure S5. Power-law scaling as a function of the $\beta = 1/T$ model parameter. **a)** We evaluated the goodness-of fit of power-law scaling of the correlation function, $g(r)$, the variance of coarse-grained variables, $V(K)$, and the eigen-spectrum, $\lambda(\text{rank}/K)$. For this, we used the ratio R_{EV} between the explained variance using the power law and the one obtained using an exponential function. Circles indicate results from the dMRI-constraint spin model; squares indicate results from the EDR-constraint spin model. The correlation function, $g(r)$, decays as a power law of distance only around the critical point (i.e., $R_{EV} > 1$ for $\beta/\beta_c \sim 1$). The variance of coarse-grained variables, $V(K)$, presents a power-law scaling for all temperatures, i.e., $R_{EV} > 1$ for all β/β_c. The power-law scaling of eigen-spectrum, $\lambda(\text{rank}/K)$, was only observed in the supercritical regime ($\beta/\beta_c < 1$) and around the critical point ($\beta/\beta_c \sim 1$). **b-d)** Eigen-spectrum, $\lambda(\text{rank}/K)$, for temperatures in the supercritical (b), critical (c), and subcritical (d) regimes using the dMRI-constraint spin model.

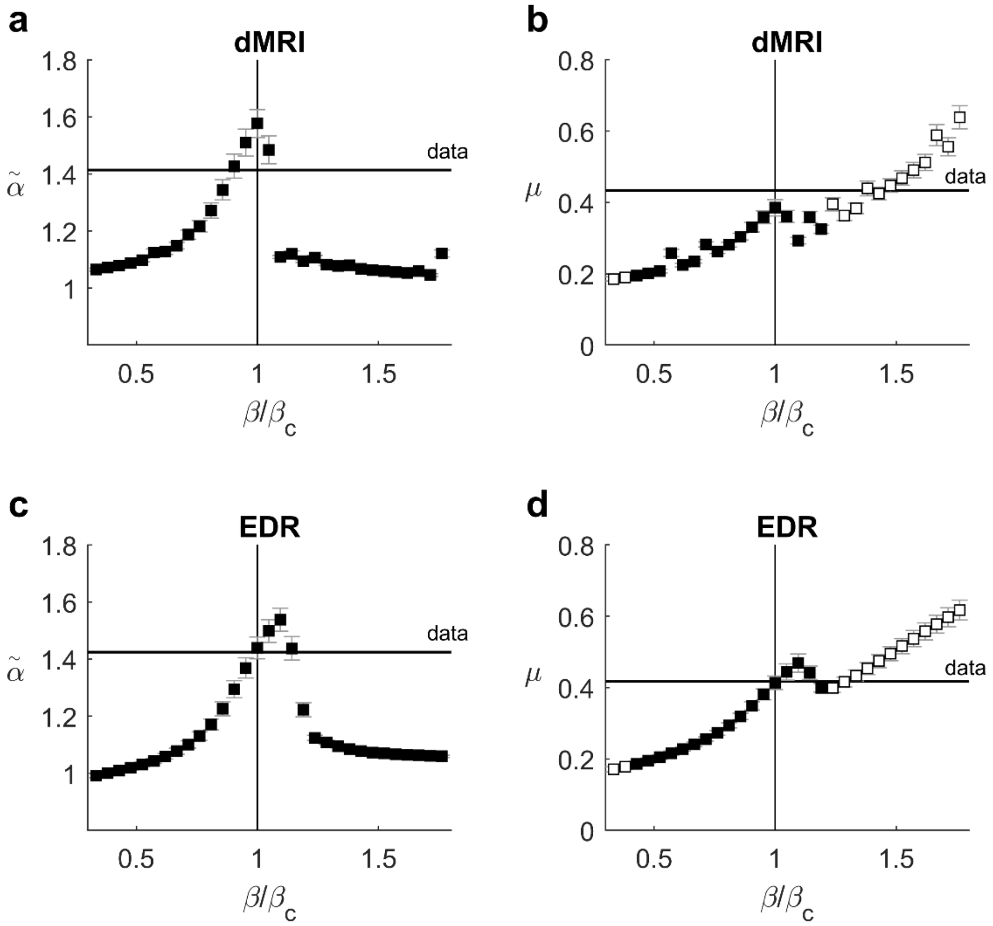

Supplementary figure S6. Connectivity-based PRG exponents of the spin model. **a-b)** Exponents $\tilde{\alpha}$ (a) and μ (b) as a function of β/β_c. The dMRI connectivity was used to simulated the spin model and to coarse-grain the activity of both the model and the fMRI data. Filled symbols indicate $R_{EV} > 1$ (favoring the power law over the exponential fit). Error bars indicate the exponent estimation error. The horizontal line indicates the empirically measured exponent. **c-d)** Same as (a) and (b), but using the EDR connectivity to simulated the spin model and to coarse-grain the activity of both the model and the fMRI data. Note that the exponents $\tilde{\alpha}$ and μ obtained from the EDR-constraint model are the closest to those observed in the data for $\beta/\beta_c = 1$.
