# Supplementary material for: Critical scaling of whole-brain resting-state dynamics
Source: Commun Biol. 2023 Jun 10;6:627. doi: 10.1038/s42003-023-05001-y (PMC10257708; doi:10.1038/s42003-023-05001-y)
Supplement: Supplementary file 3 — Description of Additional Supplementary Files [file 42003_2023_5001_MOESM3_ESM.pdf]

Description of Additional Supplementary Files

File name: Supplementary Data 1

Description: Source data behind Figure 1

File name: Supplementary Data 2

Description: Source data behind Figure 2

File name: Supplementary Data 3

Description: Source data behind Figure 3

File name: Supplementary Data 4

Description: Source data behind Figure 4

File name: Supplementary Data 5

Description: Source data behind Figure 5
