# Supplementary material for: Critical scaling of whole-brain resting-state dynamics
Source: Commun Biol. 2023 Jun 10;6:627. doi: 10.1038/s42003-023-05001-y (PMC10257708; doi:10.1038/s42003-023-05001-y)
Supplement: Supplementary file 9 — Reporting Summary [file 42003_2023_5001_MOESM9_ESM.pdf]

nature portfolio

Corresponding author(s): Adrián Ponce-Alvarez

Last updated by author(s): Feb 14, 2023

Reporting Summary

Nature Portfolio wishes to improve the reproducibility of the work that we publish. This form provides structure for consistency and transparency in reporting. For further information on Nature Portfolio policies, see our Editorial Policies and the Editorial Policy Checklist.

Statistics

For all statistical analyses, confirm that the following items are present in the figure legend, table legend, main text, or Methods section.

n/a	Confirmed	
☐	☒	The exact sample size (*n*) for each experimental group/condition, given as a discrete number and unit of measurement
☐	☒	A statement on whether measurements were taken from distinct samples or whether the same sample was measured repeatedly
☐	☒	The statistical test(s) used AND whether they are one- or two-sided<br>*Only common tests should be described solely by name; describe more complex techniques in the Methods section.*
☒	☐	A description of all covariates tested
☒	☐	A description of any assumptions or corrections, such as tests of normality and adjustment for multiple comparisons
☐	☒	A full description of the statistical parameters including central tendency (e.g. means) or other basic estimates (e.g. regression coefficient) AND variation (e.g. standard deviation) or associated estimates of uncertainty (e.g. confidence intervals)
☒	☐	For null hypothesis testing, the test statistic (e.g. *F*, *t*, *r*) with confidence intervals, effect sizes, degrees of freedom and *P* value noted<br>*Give P values as exact values whenever suitable.*
☒	☐	For Bayesian analysis, information on the choice of priors and Markov chain Monte Carlo settings
☒	☐	For hierarchical and complex designs, identification of the appropriate level for tests and full reporting of outcomes
☒	☐	Estimates of effect sizes (e.g. Cohen's *d*, Pearson's *r*), indicating how they were calculated

Our web collection on statistics for biologists contains articles on many of the points above.

Software and code

Policy information about availability of computer code

Data collection	- HCP MRI data: Van Essen et al., 2013 https://www.humanconnectome.or<br>g/study/hcp-young-adult<br>- SPM: https://www.fil.ion.ucl.ac.uk/spm/<br>- FSL: FMRIB Software Library https://fsl.fmrib.ox.ac.uk/fsl/fslwi<br>ki/<br>Connectome Workbench: Marcus et al., 2011 https://www.humanconnectome.or<br>g/software/connectomeworkbench
Data analysis	MATLAB R2021a: MathWorks https://www.mathworks.com/<br>Codes to perform the analysis and to simulate the model available at: https://github.com/adrianponce/Scaling-of-whole-brain-resting-state-dynamics.

For manuscripts utilizing custom algorithms or software that are central to the research but not yet described in published literature, software must be made available to editors and reviewers. We strongly encourage code deposition in a community repository (e.g. GitHub). See the Nature Portfolio guidelines for submitting code & software for further information.

Data

Policy information about availability of data

All manuscripts must include a data availability statement. This statement should provide the following information, where applicable:

- Accession codes, unique identifiers, or web links for publicly available datasets
- A description of any restrictions on data availability
- For clinical datasets or third party data, please ensure that the statement adheres to our policy

The data is available at https://www.humanconnectome.org/study/hcp-young-adult

Human research participants

Policy information about studies involving human research participants and Sex and Gender in Research.

Reporting on sex and gender	Sex and gender were not considered in the present study
Population characteristics	-fMRI: 1003 participants from public data release from the Human Connectome Project (HCP)<br>-dMRI: 32 participants from the HCP database
Recruitment	Data from the March 2017 public data release from the Human Connectome Project (HCP).
Ethics oversight	The Washington University–University of Minnesota (WU-Minn HCP) Consortium obtained full written informed consent from all participants to study procedures and data sharing outlined by HCP, and research procedures and ethical guidelines were followed in accordance with Washington University institutional review board approval.

Note that full information on the approval of the study protocol must also be provided in the manuscript.

Field-specific reporting

Please select the one below that is the best fit for your research. If you are not sure, read the appropriate sections before making your selection.

☒ Life sciences ☐ Behavioural & social sciences ☐ Ecological, evolutionary & environmental sciences

For a reference copy of the document with all sections, see nature.com/documents/nr-reporting-summary-flat.pdf

Life sciences study design

All studies must disclose on these points even when the disclosure is negative.

Sample size	We used all the available resting-state fMRI data, corresponding to 1003 participants from public data release from the Human Connectome Project (HCP). The dMRI data was obtained from 32 participants from the HCP database.
Data exclusions	N/A
Replication	N/A
Randomization	N/A
Blinding	N/A

Reporting for specific materials, systems and methods

We require information from authors about some types of materials, experimental systems and methods used in many studies. Here, indicate whether each material, system or method listed is relevant to your study. If you are not sure if a list item applies to your research, read the appropriate section before selecting a response.

Materials & experimental systems

n/a	Involved in the study
x	Antibodies
x	Eukaryotic cell lines
x	Palaeontology and archaeology
x	Animals and other organisms
x	Clinical data
x	Dual use research of concern

Methods

n/a	Involved in the study
x	ChIP-seq
x	Flow cytometry
	x MRI-based neuroimaging

Magnetic resonance imaging

Experimental design

Design type: Resting-state data

Design specifications: The participants were scanned on a 3T connectome-Skyra scanner (Siemens). We used one rs-fMRI acquisition of approximately 15 minutes, with eyes open and relaxed fixation on a projected bright cross-hair on a dark background. The HCP website (https://www.humanconnectome.org/) provides the details of participants, the acquisition protocol and preprocessing of the functional data.

Behavioral performance measures: N/A

Acquisition

Imaging type(s): Functional MRI; diffusion MRI

Field strength: 3T

Sequence & imaging parameters:
-Sequence: Gradient-echo EPI
-TR: 720 ms
-TE: 33.1 ms
-flip angle 52 deg
-FOV: 208x180 mm (RO x PE)
-Matrix: 104x90 (RO x PE)
-Slice thickness: 2.0 mm; 72 slices; 2.0 mm isotropic voxels
-Multiband: factor 8
-Echo spacing: 0.58 ms
-BW: 2290 Hz/Px

Area of acquisition: Whole-Brain

Diffusion MRI: [x] Used [ ] Not used

Parameters:
-Sequence: Spin-echo EPI
-TR: 5520 ms
-TE: 89.5 ms
-flip angle: 78 deg
-refocusing flip angle: 160 deg
-FOV: 210x180 (RO x PE)
-matrix: 168x144 (RO x PE)
-slice thickness: 1.25 mm, 111 slices, 1.25 mm isotropic voxels
-Multiband factor: 3
-Echo spacing: 0.78 ms
-BW: 1488 Hz/Px
-Phase partial Fourier: 6/8
-b-values: 1000, 2000, and 3000 s/mm2

Preprocessing

Preprocessing software: Standardized methods using FSL (FMRIB Software Library), FreeSurfer, and the Connectome Workbench software

Normalization: FLIRT

Normalization template: CIFTI

Noise and artifact removal: The HCP website (https://www.humanconnectome.org/) provides the full details of the processing of the data

Volume censoring	As per HCP pipeline

Statistical modeling & inference

Model type and settings	System of binary spins inetracting through structural connectivity
Effect(s) tested	Correlation function and scaling of collective activity as a function of coarse-graining

Specify type of analysis: ☒ Whole brain ☐ ROI-based ☐ Both

Statistic type for inference (See Eklund et al. 2016)	N/A
Correction	N/A

Models & analysis

n/a	Involved in the study
☐	☒ Functional and/or effective connectivity
☐	☒ Graph analysis
☐	☒ Multivariate modeling or predictive analysis

Functional and/or effective connectivity	Pearson correlations; Covariances; Covariance eigen-decomposition
Graph analysis	Node strength; Connection weight-distance relationship
Multivariate modeling and predictive analysis	Phenomenological Renormalization Group method (Meshulam et al. 2019)
